# Detection of *KMT2A* Partial Tandem Duplication by Optical Genome Mapping in Myeloid Neoplasms: Associated Cytogenetics, Gene Mutations, Treatment Responses, and Patient Outcomes

**DOI:** 10.3390/cancers16244193

**Published:** 2024-12-16

**Authors:** Qing Wei, Shimin Hu, Jie Xu, Sanam Loghavi, Naval Daver, Gokce A. Toruner, Wei Wang, L. Jeffrey Medeiros, Guilin Tang

**Affiliations:** 1 Department of Hematopathology, MD Anderson Cancer Center, The University of Texas, Houston, TX 77030, USA; qwei1@mdanderson.org (Q.W.); shu1@mdanderson.org (S.H.); jxu9@mdanderson.org (J.X.); sloghavi@mdanderson.org (S.L.); gatoruner@mdanderson.org (G.A.T.); wwang13@mdanderson.org (W.W.); ljmedeiros@mdanderson.org (L.J.M.); 2 Department of Leukemia, MD Anderson Cancer Center, The University of Texas, Houston, TX 77030, USA; ndaver@mdanderson.org

**Keywords:** *KMT2A* PTD, optical genome mapping, AML, MDS, CMML

## Abstract

*KMT2A* partial tandem duplication (PTD) involves intragenic *KMT2A* duplications and has been associated with poorer prognosis. In this study, we evaluated *KMT2A* PTD in 1277 patients with hematological malignancies using optical genome mapping (OGM). *KMT2A* PTD was detected in 35 patients with acute myeloid leukemia (AML) (7%), 5 patients with myelodysplastic syndrome (MDS) (2.2%), and 5 patients with chronic myelomonocytic leukemia (CMML) (7.1%). The PTDs varied in size, region, and copy number. An Archer RNA fusion assay confirmed *KMT2A* PTD in all 25 patients tested: 15 spanning exons 2 to 8 and 10 spanning exons 2 to 10. Most patients exhibited a normal (*n* = 21) or non-complex (*n* = 20) karyotype. The most common chromosomal abnormalities included loss of 20q or 7q and trisomy 11/gain of 11q. All patients had gene mutations, with *FLT3* ITD and *DNMT3A* prevalent in AML and *DNMT3A* and *RUNX1* common in MDS and CMML. Among patients who received treatment and had at least one follow-up bone marrow evaluation, 82% of those with de novo AML achieved complete remission after initial induction chemotherapy, whereas 90% of patients with secondary or refractory/relapsed AML showed refractory or partial responses. All but one patient with MDS and CMML were refractory to therapy. We conclude that OGM is an effective tool for detecting *KMT2A* PTD. Neoplasms with *KMT2A* PTD frequently harbor gene mutations and display normal or non-complex karyotypes. Patients with *KMT2A* PTD are generally refractory to conventional therapy, except for de novo AML.

## Simple Summary

*KMT2A* partial tandem duplication (PTD) involves intragenic duplications within the *KMT2A* gene and has been associated with acute myeloid leukemia (AML) and myelodysplastic syndrome (MDS). *KMT2A* PTD cannot be detected by conventional karyotyping or fluorescence in situ hybridization (FISH). In this study, we used optical genome mapping (OGM) to analyze the cytogenomic alterations in 1277 hematolymphoid neoplasms and identified *KMT2A* PTD exclusively in patients with myeloid neoplasms, including 35 (7%) with AML, 5 (2.2%) with MDS, and 5 (7.2%) with chronic myelomonocytic leukemia (CMML). Neoplasms with *KMT2A* PTD frequently exhibit a normal or non-complex karyotype and consistently harbor gene mutations involving epigenetic regulators, the *FLT3/RAS* signaling pathway, transcription factors, and spliceosome genes. Patients with *KMT2A* PTD are generally resistant to conventional chemotherapy, with the exception of those with *de novo* AML, which demonstrates a high remission rate. Patients with KMT2A PTD positive secondary AML and MDS had a poor outcome.

## 1. Introduction

*KMT2A* (also known as *MLL*), located on chromosome 11q23, is frequently rearranged in pediatric and adult leukemias across different lineages [1]. Unlike *KMT2A* fusion events, *KMT2A* partial tandem duplication (PTD) is an intragenic in-frame duplication within the N-terminal region of *KMT2A*. The breakpoints often occur in flanking intronic sequences of exons 2 to 8 or 10 and are often facilitated by Alu elements [2,3,4]. This alteration occurs in approximately 5–10% of cases of acute myeloid leukemia (AML) [2] and 3–5% of myelodysplastic syndromes (MDS) [5]. *KMT2A* PTD is often associated with AML with a normal karyotype and is enriched in AML with trisomy 11 as a sole chromosome aberration [4]. Studies suggest that *KMT2A*-PTD alone is insufficient to cause AML and additional genetic aberrations are required for the development of *KMT2A* PTD-associated leukemia [6,7]. Multiple studies have shown that *KMT2A* PTD AML is often associated with *FLT3* ITD and the *RAS* signaling pathway. It frequently co-occurs with other mutations in genes of epigenetic regulators (e.g., *DNMT3A* and *IDH1*/*IDH2*), transcription factors (e.g., *RUNX1*), and spliceosome genes (e.g., *U2AF1* and *SRSF2*) [8,9,10].

The prognosis impact of *KMT2A* PTD AML remains controversial [11]. In MDS, *KMT2A*-PTD is associated with excess blasts, a high risk of AML transformation, and poorer overall survival [5,12,13]. It is recognized as a high-risk molecular feature in the International Prognostic Scoring System-Molecular (IPSS-M) [12]. To date, the study of *KMT2A* PTD in other hematological neoplasms is scarce, including chronic myelomonocytic leukemia (CMML).

*KMT2A* PTD has been shown to drive HOX overexpression in both mouse models and AML with *KMT2A* PTD [14,15], suggesting potential therapeutic implications, particularly for the use of menin inhibitors [16]. At our institution, we are conducting a phase 2 study of revumenib for relapsed/refractory (R/R) disease targeting high HOX expression, including cases with *KMT2A* PTD. Detecting *KMT2A* PTD may help identify patients eligible for menin inhibitor therapies.

Detecting *KMT2A* PTD is challenging because it is a cryptic intragenic alteration that is not detectable by conventional karyotyping or fluorescence in situ hybridization (FISH) analysis [3]. Additionally, these intragenic duplications exceed the amplification capacity of traditional polymerase chain reaction (PCR) strategies. Furthermore, a recent report described rare AML cases with both *KMT2A* rearrangement and *KMT2A* PTD, where *KMT2A* rearrangement likely represented the founder clone, further complicating the detection of *KMT2A* PTD [17]. Historically, methods such as Southern blot hybridization [18,19], reverse transcription (RT) PCR, complementary cDNA sequencing [2,3], and cytogenomic microarray [13] have been employed, but there is a growing interest in employing DNA- or RNA-based next-generation sequencing (NGS) assays for routine clinical testing to detect *KMT2A* PTD [20,21,22].

Optical genome mapping (OGM) has emerged as a novel technology that enables high-resolution, comprehensive analysis of the whole genome, including intragenic gene duplications. To date, only one study has used OGM as an orthogonal method to confirm *KMT2A* PTD during the validation of a DNA-based NGS assay in a limited number of cases; the results suggested that OGM may be a superior tool for detecting *KMT2A* PTD compared to NGS [23].

In this study, we used OGM to evaluate the cytogenomic changes in a large cohort of patients with hematolymphoid tumors. *KMT2A* PTD was detected exclusively in myeloid neoplasms, including AML, MDS, and CMML. We analyzed the cytogenetic and molecular profiles of these neoplasms and further assessed the patients’ associated clinical characteristics, treatment responses, and outcomes.

## 2. Materials and Methods

### 2.1. Patients

We reviewed hematologic malignancies tested by OGM at our institution between 1 November 2022 and 15 October 2024 and collected the cases that showed *KMT2A* PTD. Of note, a part of these patients have been published previously [24]. Clinical information was retrieved from the electronic medical records. This study was approved by the Institutional Review Board of MD Anderson Cancer Center and was conducted in accord with the Declaration of Helsinki.

### 2.2. Conventional Chromosome Analysis

G-banded chromosomal analysis was performed as part of our routine diagnostic workup for hematological malignancies. Twenty Giemsa-banded metaphase cells were analyzed, and the results were reported using the International System for Human Cytogenetic Nomenclature (ISCN 2020). A complex karyotype was defined as ≥3 unrelated chromosomal abnormalities with at least one structural abnormality.

### 2.3. Optical Genome Mapping

**Figure 1:**
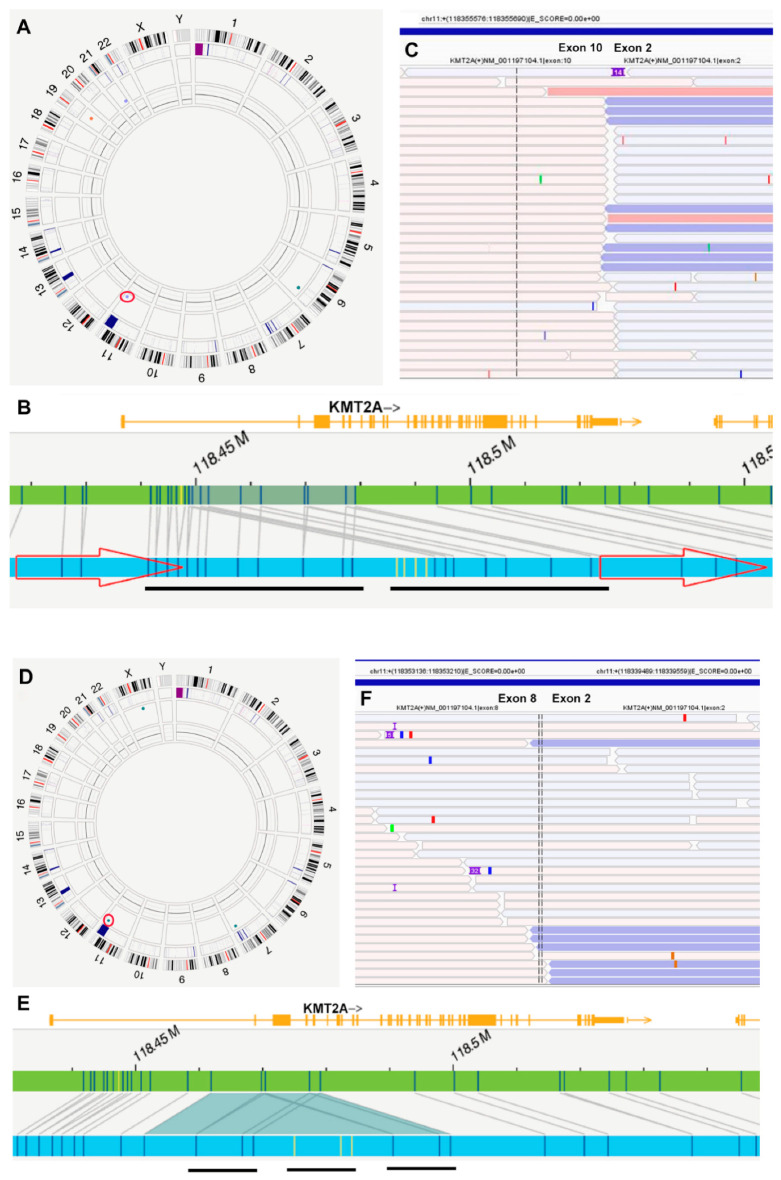
Detection of *KMT2A* PTD by optical genome mapping (OGM) and Archer RNA fusion assay. A–C (Case #18): The OGM Circos plot (**A**) indicates a duplication (circled) on chromosome 11q23, with detail provided in Genome Browser (**B**). OGM identified this as dup(11)(q23.3q23.3), with coordinates RefStartPos 118,448,714 and RefEndPos 118,479,068, size 30,354 bp, and a VAF of 0.79. The duplicated sequence, verified by manual examination, is underlined. The RNA fusion assay demonstrates a fusion between exon 10 and exon 2 of KMT2A (**C**). (**D**–**F**) (Case #5): The OGM Circos plot (**D**) shows an insertion (circled), ins(11q23;?), on chromosome 11q23, with detail provided in the Genome Browser (**E**). The “unknown” inserted sequence, marked in yellow by OGM, has coordinates RefStartPos 118,461,867 and RefEndPos 118,479,068, size 31,007 bp, and a VAF of 0.78. Manual examination reveals an “insertion” and duplication of the same fragment, resulting in a triplication. The triplicated sequence is underlined. The RNA fusion assay shows a fusion between exon 8 and exon 2 of *KMT2A* (**F**).

OGM was performed on fresh peripheral blood or bone marrow aspirate specimens following the manufacturer’s protocol (Bionano Genomics, San Diego, CA, USA), as previously described [24]. In brief, ultra-high-molecular-weight genomic DNA (UHMW gDNA) was extracted from approximately 1.5 million cells and labeled using the Bionano Prep Direct Label and Stain (DLS) kit, which tags DNA with a fluorescent label and stains the DNA backbone. The labeled gDNA was imaged using the Bionano Saphyr, acquiring approximately 1500 Gb of DNA per sample. DNA molecules were then assembled into a genome with >400× coverage using the Bionano Solve software (version Solve3.8.2). Data analysis was performed using the Rare Variant Analysis Pipeline in Bionano Access (version 1.8.2), focusing on clinically relevant genes and loci based on the HemeTargets and hg38-primary_transcripts feature files [24]. *KMT2A* PTD was identified as an intragenic insertion and/or intragenic duplication of *KMT2A*, based on the patterns shown in Figure 1 and Figure S1. Although OGM identified an insertion, ins(11;?), in most cases, manual examination revealed that these insertions were duplications or triplications of genic fragments within *KMT2A*.

### 2.4. Next-Generation Sequencing

DNA-based NGS for somatic mutations was performed on all cases as part of our routine diagnostic workup, targeting all exonic or “hotspot” regions of 81 genes commonly mutated in myeloid neoplasms, as previously described [24,25].

### 2.5. FLT3 ITD and TKD Analysis

A multiplex fluorescent-based PCR analysis followed by capillary electrophoresis was performed to detect *FLT3* internal tandem duplication (ITD) and/or tyrosine kinase domain (TKD) mutations on DNA isolated from BM aspirate samples, as described previously [26].

### 2.6. Archer RNA Fusion Assay

The EndLeukemia RNA Translocation Assay is an NGS-based analysis interrogating 106 genes with the ability to detect targeted inter- and intragenic translocations using RNA extracted from peripheral blood or bone marrow aspirate specimens. Target enrichment is achieved through anchored multiplex PCR (AMP), and sequencing is performed bidirectionally on an Illumina sequencer. The resulting sequencing reads are aligned with the human reference genome GRCh37/hg19, and structural variants are identified using Archer Analysis Software v6.2.7. The analytical sensitivity of this assay is estimated at 100% in validation studies involving samples with at least 5% disease involvement. This assay detects *KMT2A* PTD as an abnormal *KMT2A* exonic junction between upstream and downstream exons.

## 3. Results

### 3.1. Patients

A total of 1277 patients with hematolymphoid tumors were evaluated by OGM during the study interval, including 855 patients with myeloid neoplasms and 422 patients with B- and T-cell neoplasms. The myeloid neoplasms included 502 AML, 228 MDS, 38 myeloproliferative neoplasms (MPN), and 87 MDS/MPN (including 70 CMML). *KMT2A* PTD was detected in 45 patients: 35 (7%) with AML, 5 (2.2%) with MDS, and 5 (7.1%) with CMML. The diagnosis and demographic features of the patients with *KMT2A* PTD are summarized in Table 1. There were 31 men and 14 women, with a median age of 68 years (range, 23–86). Among the AML patients, 14 were newly diagnosed (de novo), 14 were refractory/relapsed (R/R), and 7 had a prior history of myeloid neoplasm (secondary AML, sAML). The sAML cases were preceded by MDS (*n* = 5), CMML (*n* = 1), and polycythemia vera (*n* = 1) (Table 1). Of note, 11 (31%) AML cases showed myelomonocytic or monoblastic differentiation.

### 3.2. Detection of KMT2A PTD by OGM and Confirmation by Archer RNA Fusion Assay

OGM detected an intragenic “insertion” of *KMT2A* in 41 cases and intragenic duplications of *KMT2A* in 4 cases, both corresponding to *KMT2A* PTD (Table 2, Figure 1, Figure S1 and Table S1). Although OGM often called these events an “insertion” of unknown material, ins(11;?)(q23.3;?), most cases were recognized as repeats of sequence based on the label patterns by manual review. A manual review showed that the PTD spanned from intron 1, exon 2, or intron 2 to intron 5 (or exon 6) in all cases. The number of repeats ranged from two to four (two repeats in 26 cases, five in 17 cases, and four in 2 cases) (Figure 1 and Figure S1 and Table S1). The variant allele frequency (VAF) ranged from 0.37 to 0.93, with a median of 0.82. Based on the VAF provided by OGM, the number of repeats (manually verified), and blast counts (used as a proxy for neoplastic cells or clonal size) in AML, the estimated fractional copy number of these “duplicated” regions ranged from 3 to 17.2. No significant differences in fractional copy number of PTD were observed among patients with de novo AML, R/R AML, or sAML. Due to uncertainty about clonal size in MDS and CMML, estimating the fraction copy number of PTD in these conditions was challenging.

RNA fusion assays were performed on 25 cases, all showing *KMT2A* PTD: 15 showed PTD spanning from exon 2 to exon 8 (chr11:118,353,210::118,339,490), and 10 cases from exon 2 to exon 10 (chr11:118,355,690::118,339,490) (Figure 1). The discrepancy between OGM and the RNA Anchor assay at the exon level is primarily due to the absence of labeling sites between exons 6 and 16 of KMT2A in OGM (Figure S3). Additionally, KMT2A PTD was identified by RNA sequencing in one case (#29) at an outside hospital (Table 2).

### 3.3. Cytogenomic Abnormalities Detected by G-Banded Karyotyping and OGM

Among 44 patients with an available karyotype, 21 (48%) had a normal karyotype, 20 (45%) exhibited 1–2 abnormalities, and 3 (7%) had a complex karyotype. The complex karyotype cases included two AML patients: case #15 with a prior history of polycythemia vera and case #17 with a history of mantle cell lymphoma who developed therapy-related MDS and later progressed to AML. A third patient (#38) had refractory MDS with 17% blasts.

OGM identified *KMT2A* PTD as a sole abnormality in 20 patients and with additional cytogenomic abnormalities in 25 patients (Table 2). Additional structural variants (SVs) were detected in six patients, including intragenic insertions of *RUNX1* ins(21;?)(q22.12;?) in two patients (#17, #37). Other SVs were found in single cases, such as chromoanagenesis in case #15. Copy number variants (CNVs) were detected in 23 cases, with loss of 20q (*n* = 7) or 7q (*n* = 6) and trisomy 11/gain of 11q (+11/+11q, *n* = 6) being the most common. Other recurrent CNVs included del(5q), trisomy 8, trisomy 13, del(9q) and del(12q), detected in two or more cases.

### 3.4. Gene Mutations and FLT3 ITD

**Figure 2:**
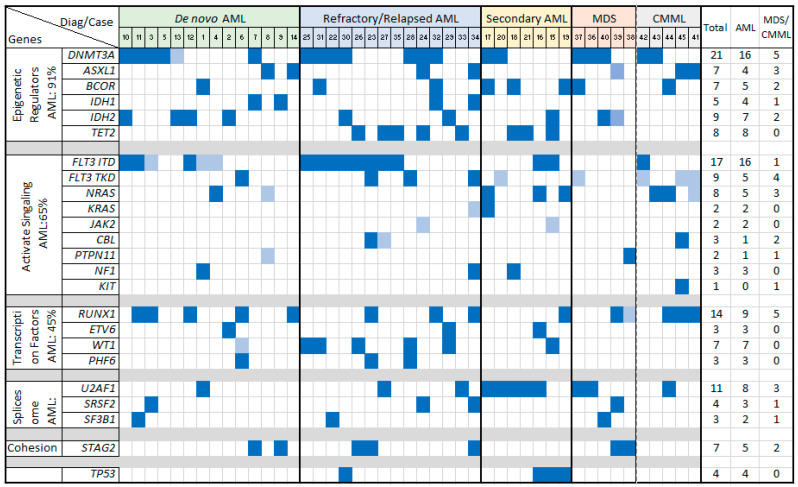
Gene mutations in neoplasms with *KMT2A*-PTD, sorted by variant categories. Fills in dark blue: VAF ≥ 0.05; fills in light blue: VAF < 0.05.

In the AML group, the most frequently affected pathways and genes included the following: epigenetic regulators (e.g., *DNMT3A* and *IDH1/IDH2*), observed in 91% of cases; the *FLT3-RAS* pathway (e.g., *FLT3* ITD) in 65% of cases; transcription factors (e.g., *RUNX1*) in 45% of cases; spliceosome genes (e.g., *U2AF1* and *SRSF2*) in 37% of cases; and cohesion genes (e.g., *STAG2*) in 20% of cases. Specifically, *FLT3* ITD mutations were identified in 16 cases, while *FLT3* TKD mutations appeared in 3 cases, including 1 case (#23) with both mutations. The *FLT3* ITD ratio and VAF were notably higher in R/R AML compared to de novo and sAML cases. No *TP53* mutations were detected in de novo AML, but these mutations were present in three sAML cases (#15, #16, and #19) and one R/R AML case (#30). Similarly, *TET2* mutations were not detected in de novo AML but were presented in two sAML and five R/R AML cases (Table 3, Figure 2).

In the MDS group, the most commonly affected pathways involved epigenetic regulators (e.g., *DNMT3A*) and spliceosome genes (e.g., *U2AF1*), observed in four out of five cases. Notably, one MDS case with a proliferative mutation in *PTPN11* progressed to AML. In the CMML group, recurrently altered pathways included epigenetic regulators (e.g., *DNMT3A*) and *FLT3* TKD/*RAS* kinase mutations, with all five cases showing these mutations. *FLT3* TKD was more prevalent than *FLT3* ITD in both MDS and CMML, although both mutations appeared as subclonal with low VAFs (Table 2, Figure 2).

### 3.5. Response to Treatments and Outcomes

Among the 35 patients with *KMT2A* PTD AML, 31 received chemotherapy, with some also receiving FLT3 or IDH2 inhibitors as part of their mainline treatments (Table 1).

Of the 14 patients with de novo AML, 11 received treatment and had follow-up. Nine patients responded well to treatments and achieved remission, while two showed a partial response. Five patients subsequently underwent allogeneic stem cell transplant (allo-SCT). By the end of the follow-up period, three patients had died (two from bone marrow failure and one from unknown causes), eight were alive in complete remission, two were still receiving induction chemotherapy, and one was lost to follow-up. The median overall survival (OS) was not reached.

Among the 14 patients with R/R AML, 9 were refractory to therapy, 3 exhibited partial response to treatments, and 2 responded well to treatments and achieved remission. By the end of follow-up, seven patients had died: three with persistent AML, two in remission, and two with no follow-up after the detection of *KMT2A* PTD, with a median OS of 19.2 months from diagnosis or 5.3 months from the detection of *KMT2A* PTD.

Of the seven patients with secondary AML, four were refractory and three showed partial response to treatments. By the end of follow-up, four had died and three had persistent AML, with a median OS of 4.5 months.

Among the five patients with MDS associated with *KMT2A* PTD, one patient died within a month of diagnosis due to multiple comorbidities. The remaining four patients received hypomethylating regimens, but all were refractory to treatment: one patient progressed to AML, and the other three had persistent disease. By the end of the follow-up period, three patients had died, and two were alive with persistent disease. The median overall survival was 8.5 months.

All five patients with CMML associated with *KMT2A* PTD received treatments. One patient (#41) responded to a combination of decitabine and venetoclax, followed by allo-SCT, and was in remission by the end of the follow-up. One patient (#43) progressed to AML and the other three were refractory to treatments. By the end of the follow-up, one patient had died, one was in remission, two patients had persistent CMML, and one had AML. The median overall survival was not reached.

## 4. Discussion

In this study, we used OGM to identify *KMT2A* PTD across a spectrum of hematological neoplasms. *KMT2A* PTD was detected exclusively in a subset of myeloid neoplasms, including AML, MDS, and CMML. OGM provides straightforward visualization of *KMT2A* PTD and is a reliable tool for detecting *KMT2A* PTD. Among these 45 cases, 25 were assessed using the Archer RNA fusion assay, and 1 was assessed by RNA sequencing. *KMT2A* PTD was confirmed in all 26 cases, demonstrating 100% concordance with RNA fusion assays. Conversely, cases that did not show *KMT2A* PTD by OGM were also negative by RNA fusion assays in those cases both assays were performed. In a previous study [23], *KMT2A* PTD was detected in 41 of 932 (4.4%) AML cases by using DNA-based NGS; of these 41 patients, 13 were also tested by OGM. OGM confirmed *KMT2A* PTD in 11 cases, whereas 2 cases initially shown by NGS to have *KMT2A* PTD were proven to be *KMT2A* rearranged by OGM. These findings underscore the high sensitivity and specificity of OGM for *KMT2A* PTD detection. Nonetheless, we observed certain limitations with OGM, including challenges in estimating PTD size, exon involvement, PTD copy number, and clarifying the complexity of PTD.

Most of the cases in this cohort had a normal or non-complex karyotype. Trisomy 11 or +11q is an uncommon cytogenetic abnormality in myeloid neoplasms [27]; however, it is frequently observed in cases with *KMT2A* PTD; approximately 30% to 70% of myeloid neoplasms with +11/+11q cases harbor *KMT2A* PTD [19,28,29,30]. During this study period, we identified 15 myeloid neoplasms with +11/+11q, and 6 (40%) had *KMT2A* PTD. Conversely, *KMT2A* PTD appeared to be mutually exclusive with class-defining chromosomal or gene rearrangements, as has been reported in other studies [28,29]. Deletions of 7q and 20q are common cytogenetic abnormalities in myeloid neoplasms and were likewise observed frequently in cases with *KMT2A* PTD [29]. The additional cytogenetic abnormalities did not appear to affect patient survival in this cohort, as has been reported by others [30].

Gene mutations are very common in myeloid neoplasms with *KMT2A* PTD, detected in all (100%) cases in this cohort and in a similarly high percentage of cases in another study [30]. The frequent co-occurrence of additional mutations appears critical for leukemogenesis in *KMT2A* PTD. Mouse model studies have shown that *KMT2A* PTD alone is insufficient to induce leukemia [6]. For instance, *DNMT3A* mutations enhance the self-renewal capacity of *KMT2A* PTD-positive leukemic cells, conferring a proliferative advantage [31]. Furthermore, mice with both *KMT2A* PTD and *FLT3 ITD* develop acute leukemia, highlighting the role of cooperative mutations in leukemogenesis [6].

The most frequently mutated genes included *DNMT3A*, *FLT3-ITD*, *RUNX1*, *U2AF1*, *STAG2*, *TET2*, and *IDH2*; no cases carried *NPM1* mutation. These results are in accord with other reports [9,10,28,30]. *DNMT3A*, the most frequently mutated gene, exhibited hotspot mutations (R882H/C) and stop-gain mutations, mirroring earlier observations. Recurrent hotspot mutations in *IDH1* (R132C), *IDH2* (R140Q), *U2AF1* (S34F), and *FLT3* (D835Y/V/E) are suggestive of gain-of-function effects. Conversely, mutations in genes such as *ASXL1*, *TET2*, *RUNX1*, *WT1*, *STAG2*, and *PHF6* were predominantly frameshift or stop-gain mutations, indicative of loss-of-function mechanisms. Additionally, mutually exclusive relationships between mutations in *IDH1/IDH2* and *TET2*, as well as *STAG2* and *U2AF1*, were observed in our cohort, consistent with earlier studies [10].

The association of these gene mutations with patient overall survival (OS) remains controversial. Some investigators have found that *DNMT3A* mutations, especially non-R882 variants, correlate with shorter OS [9,21,28,30]. In our cohort, although patients with *DNMT3A* mutations had a slightly shorter OS compared to those without (12.4 vs. 15.6 months), this difference was not statistically significant. We also observed that mutations in *TP53* and *TET2* were not detected in de novo AML but presented in sAML and R/R AML, suggesting that these mutations were likely acquired during disease relapse or progression and may influence treatment response and outcomes.

Patients with de novo AML and *KMT2D* PTD generally show a good response to treatment, with a high (70~90%) remission rate after the first induction in this cohort and others reported previously [18,19]. But these patients are reported to experience a high relapse rate, up to 70% with a median disease-free survival of 7.75 months in one study [18]. In another study, these patients had a 100% relapse rate within a year [19]. In the de novo AML group of this study, no relapses were observed, which could be attributable to the relatively short follow-up time, different therapeutic regimens compared to previous studies, and the fact that more patients received allo-SCT after the first remission. Although *KMT2A* PTD in AML has been associated with poorer survival, it is not generally considered an independent prognostic factor [30,32]. Some factors may impact the survival of *KMT2A* PTD AML, such as the *DNMT3A* mutations mentioned above. Whether the clonal burden of *KMT2A* PTD or copy number of PTD contributes to survival or not remains controversial. One study of AML patients found that a high initial clonal burden of *KMT2A* PTD was the only independent factor influencing remission rates and clinical outcomes [33]; however, another study did not observe this effect [21]. In the de novo AML group in this study, three patients (#3, #7, and #10) died; all three patients showed *DNMT3A* mutations and had high PTD copy numbers (estimated to be 17, 8.6, and 9.7, respectively), although the sample size is small.

Studies have shown that *KMT2A* PTD in MDS is associated with inferior survival and a high risk of progression from MDS to sAML [5,12,20]. In our cohort, patients in both the MDS and sAML groups exhibited a high rate of treatment resistance (nearly 100%) and short survival. Studies have shown that *KMT2A* PTD can be acquired during transformation from MDS to AML [5]. MDS cases also have a lower copy ratio burden of *KMT2A* PTD compared to AML [20]. In the current study, we evaluated *KMT2A* PTD at a single time point, so it is unknown whether *KMT2A* PTD was acquired during disease progression. However, *KMT2A* PTD was detected more frequently in sAML, 7 of 26 (27%) cases, as compared with AML as a whole group (7%).

Studies on *KMT2A* PTD in CMML are very limited. In this cohort we report, *KMT2A* PTD was present in approximately 7% of CMML patients, a frequency similar to that observed in AML. Four of these five patients had *KMT2A* PTD as the sole cytogenetic abnormality, but all patients showed two or more gene mutations, most commonly involving *RUNX1*, *DNMT3A*, *ASXL1*, and *NRAS* (found in 2–3 cases). This group of patients tended to have high blast counts and was frequently refractory to treatment. However, further study is needed to clarify the associated clinicopathological features and outcomes due to the relatively small number of cases and short follow-up period.

## 5. Conclusions

OGM effectively detects *KMT2A* PTD, showing 100% concordance with RNA fusion assays when both assays were performed. *KMT2A* PTD is exclusively found in myeloid neoplasms, including AML, MDS, and CMML. Patients with *KMT2A* PTD-positive MDS and secondary AML seem to often show resistance to treatment and have poorer survival. Further studies are needed to better understand the clinical course and outcomes for patients with *KMT2A* PTD-positive CMML.

## Figures and Tables

**Table 1:** Demographic and clinicopathological features of neoplasms with *KMT2A* PTD.

Case	Sex/Age	Diagnosis	BM Blasts	Main Treatment	Response to Treatments	SCT	FU Time (Mon) *	FU Time (Mon) **	Last FU
1	M/68	de novo AML	73%	CLIA + Ven	CR	Yes	12.3	12.3	ACR
2	F/40	de novo AML	60%	CLIA + Ven	CR		5.1	5.1	Alive (loss of FU)
3	M/81	de novo AML	88%	IMGN632 + AZA + Ven	Responded, BM aplasia		3.6	3.6	Died
4	M/45	de novo AML	30%	7 + 3 plus Midostaurin	CR	Yes	8.1	8.1	ACR
5	F/59	de novo AML	52%	CLIA +Ven	Partial response		2.9	3.0	ACR
6	F/44	de novo AML	35%	CLIA + Ven	CR		5.7	5.6	ACR
7	F/62	de novo AML	72%	NA	NA		0.4	0.4	Died
8	F/58	de novo AML	53%	CLIA + Ven	CR		3.0	3.0	ACR
9	M/65	de novo AML	53%	CLIA + Ven	CR		4.8	4.8	ACR
10	M/63	de novo AML	53%	DAC + Ven + Quiz	Responded, BM aplasia	Yes	9.7	9.7	Died
11	M/64	de novo AML	45%	DAC + Ven + Quiz	Partial response	Yes	5.4	5.4	ACR
12	F/69	de novo AML	73%	DAC + Ven + Quiz	CR	Yes	12.7	12.7	ACR
13	M/75	de novo AML	48%	AZA + Ven	NA		0.7	0.7	Alive (no FU BM)
14	M/71	de novo AML	37%	Clad + Ldac + Ven	NA		0.5	0.5	Alive (no FU BM)
15	M/80	sAML	84%	CLAD + LDAC + enasidenib	Partial response		4.0	3.8	Died
16	M/69	sAML	47%	AZA + Ven + Gilt	Refractory		2.2	2.2	Died
17	M/84	sAML	35%	AraC + Ven	Refractory		4.5	4.2	Died
18	M/84	sAML	42%	ASTX727 + Ven	Partial response		6.0	6.0	AWD
19	M/65	sAML	89%	Many	Refractory	Yes+	21.0	2.1	Died
20	M/78	sAML	48%	DAC + Ven + Quiz	Refractory		1.6	1.6	AWD
21	M/75	sAML	20%	Clad + Ldac + Ven	Partial response		1.5	1.5	AWD
22	M/74	R/R AML	69%	AZA + DAC + Ven + Gilt	Refractory		24.6	8.5	AWD
23	F/52	R/R AML	95%	Many	Refractory	Yes+	19.2	0.9	Died
24	M/59	R/R AML	83%	Aza	Refractory		3.2	0.0	Alive (loss of FU)
25	M/71	R/R AML	46%	ASTX727 + Ven + Gilt	CR	Yes	15.8	15.8	ACR
26	M/53	R/R AML	83%	CLIA + Gilt	CR	Yes	9.6	4.6	ACR
27	M/73	R/R AML	90%	DAC + Ven	Refractory		15.0	3.3	Died
28	F/64	R/R AML	89%	DAC + Ven + Gilt	Partial response		8.9	7.4	AWD
29	M/29	R/R AML	89%	multiple, including CLIA + Ven	Refractory	Yes+	13.9	5.3	Died
30	F/72	R/R AML	87%	DAC + Ven + Enasidenib	Refractory		11.4	3.8	Died
31	M/68	R/R AML	92%	Aza + Ven, CLAG + Gilt	Partial response		12.4	2.4	Died
32	M/23	R/R AML	24%	Multiple	Refractory	Yes+	29.8	7.9	Died
33	M/63	R/R AML	10%	CLAG	Refractory		13.3	0.0	Alive (loss of FU)
34	F/79	R/R AML	88%	7 + 3; ASTX727 + Ven	Refractory		15.6	1.0	Died
35	F/69	R/R AML	84%	AZA + Ven	Partial response		16.3	7.5	AWD
36	M/70	MDS	4%	NA	NA		1.1	1.1	Died
37	M/71	MDS, R/R	3%	ASTX030	Refractory		9.1	6.0	AWD
38	F/66	MDS, R/R	17%	Aza	Refractory, progress to AML	Yes+	19.5	8.5	Died
39	M/71	MDS, R/R	9%	Aza, DAC	Refractory		14.5	4.8	Died
40	F/65	MDS, R/R	2%	ASTX030	Refractory		23.0	1.9	AWD
41	M/73	CMML	13%	DAC + Ven	Good response	Yes	7.2	5.8	ACR
42	F/46	CMML	10%	Clad + Ldac + Gilt	Refractory		5.0	5.0	AWD
43	M/55	CMML	18%	Ven + Clad + LDAC + Aza	Refractory, prog to AML	Yes	19.3	19.3	AWD
44	M/86	CMML	2%	DAC	Refractory		6.6	6.0	AWD
45	M/65	CMML	5%	DAC + Ven	Refractory	Yes	5.3	4.4	Died

* from the time of diagnosis; ** from the time at *KMT2A* PTD detection; + allogeneic stem cell transplant (SCT) before *KMT2A* PTD detection. ACR: alive with complete remission; AML: acute myeloid leukemia; AWD: alive with disease; CMML: chronic myelomonocytic leukemia; CR: complete remission; FU: follow-up; MDS: myelodysplastic syndrome; sAML: secondary AML; R/R: refractory/relapsed. AraC: cytarabine; AZA: azacitidine (Vidaza); Clad: cladribine; DAC: decitabine; Gilt: gilteritinib; LDAC: low-dose cytarabine; Quiz: quizartinib; VEN: venetoclax; 7 + 3: 7 days of cytarabine (AraC) and 3 days of daunorubicin; ASTX030: cedazuridine + azacitidine; ASTX727: cedazuridine + decitabine; CLAG: cladribine + cytarabine + G-CSF; CLIA: cladribine + idarubicin + cytarabine.

**Table 2:** Karyotype and optical genome mapping (OGM) findings. CNV: copy number variant; SV: structural variant. Please see more detailed information on structural variants in Table S1.

Case	Karyotype	CNV by OGM	SV by OGM	Archer RNA
1	46,XY,der(7)t(7;11)(q22;q13)[6]/46,XY [14]	7q22.1q36.3(101111770_157627636)x1 11q13.4q25(72147563_135069565)) × 3	ins(11;?)(q23.3;?)/ KMT2A t(7;11)(q22.1;q13.4)	NA
2	46,XX,del(9)(q13q22),del(12)(p11.2)[2]/ 46,XX [18]	No	ins(11;?)(q23.3;?)/ KMT2A	NA
3	47,XY, +13[11]/94,idemx2[3]/46,XY [6]	(13) × 3	ins(11;?)(q23.3;?)/ KMT2A	exon8−exon2
4	46,Y,t(X;3)(q28;q21)[20]	5q35.2q35.3(176056006_178036827) × 1	ins(11;?)(q23.3;?)/ KMT2A t(X;3)(q28;q21.3)/GATA2::BRCC3	NA
5	46,XX [20]	No	ins(11;?)(q23.3;?)/ KMT2A	exon8−exon2
6	46,XX [20]	(9p24.1p23) × 1	ins(11;?)(q23.3;?)/ KMT2A	exon8−exon2
7	46,XX [20]	No	ins(11;?)(q23.3;?)/ KMT2A	exon8−exon2
8	46,XX [20]	No	ins(11;?)(q23.3;?)/ KMT2A	exon10−exon2
9	47,XY, +11[19]/46,XY [1]	(11) × 3	ins(11;?)(q23.3;?)/ KMT2A	exon10−exon2
10	47,XY, +11[20]	(11) × 3	ins(11;?)(q23.3;?)/ KMT2A	NA
11	46,XY [20]	No	ins(11;?)(q23.3;?)/ KMT2A	exon8−exon2
12	46,XX [20]	No	ins(11;?)(q23.3;?)/ KMT2A	NA
13	47,XY, +11[17]/46,XY [3]	(11) × 3	ins(11;?)(q23.3;?)/ KMT2A	exon8−exon2
14	46,XY [20]	No	ins(11;?)(q23.3;?)/ KMT2A	exon8−exon2
15	46,XY,add(5)(q13),add(7)(q31),add(11)(p15)[13]/45~46,idem,del(12)(q14),add(15)(q22)[cp7]	5q13.3q35.3(76241632_181472714) × 1 7q22.1q36.3(102532017_157625834) × 1 11q13.4q25(73229466_135069565) × 3~4	ins(11;?)(q23.3;?)/ KMT2A (7, 9, 12)cx	NA
16	NA	(9q21.13q31.1) × 1	ins(11;?)(q23.3;?)/ KMT2A	NA
17	47,XY, +8,del(12)(p13p11.1),del(20)(q11.2q13.3)[20]	6p25.3p23(76216_14959181)x1 (8) × 3 9q22.32q34.2(95217699_133526602) × 3 12p13.33p11.1(14568_34717946) × 1 16q11.1q24.3(38277017_90079974) × 1 20q11.22q13.31(33803800_56426222) × 1	ins(11;?)(q23.3;?)/ KMT2A ins(21;?)(q22.12;?)/ RUNX1	NA
18	46,XY [20]	No	ins(6;?)(q23.3;?)/ MYB dup(11)(q23.3q23.3)/ KMT2A dup(21)(q22.2q22.2)/ ERG	exon10−exon2
19	46,XY [20]	No	ins(11;?)(q23.3;?)/ KMT2A	exon10−exon2
20	46,XY,der(7)t(7;11)(q11.2;q13)[20]	No	ins(11;?)(q23.3;?)/ KMT2A dup(21)(q22.2q22.2)/ ERG	exon10−exon2
21	46,XY,del(20)(q11.2q13.1)[20]	20q11.21q13.13(32611202_50826425) × 1	ins(11;?)(q23.3;?)/ KMT2A	exon10−exon2
22	46,XY,del(9)(q21q33)[18]/46,XY [2]	9q21.11q33.3(66694956_124441888) × 1	ins(11;?)(q23.3;?)/ KMT2A	exon10−exon2
23	46,XX,del(6)(p25p22)[20]	6p25.2p22.1(3099106_30367222) × 1	ins(11;?)(q23.3;?)/ KMT2A	exon10−exon2
24	46,XY [20]	No	ins(11;?)(q23.3;?)/ KMT2A	NA
25	46,XY [20]	No	ins(11;?)(q23.3;?)/ KMT2A	NA
26	46,XY [20]	No	ins(11;?)(q23.3;?)/ KMT2A	exon8−exon2
27	48,XY, +4, +13[20]	(4) × 3 (13) × 3	ins(11;?)(q23.3;?)/ KMT2A	NA
28	46,XX [20]	No	ins(11;?)(q23.3;?)/ KMT2A	exon8−exon2
29	46,XY,t(7;10)(p22;q22)[3]/46,XY [17]	No	ins(11;?)(q23.3;?)/ KMT2A	RNA sequencing
30	46,XX [20]	No	ins(11;?)(q23.3;?)/ KMT2A	NA
31	46,XY [20]	No	ins(11;?)(q23.3;?)/ KMT2A	exon8−exon2
32	47,XY, +8[3]/46,XY,r(7)[2]/46,XY [15]	7q11.21q36.3(17599785_157009697) × 1 (8) × 3	dup(11)(q23.3q23.3)/ KMT2A	NA
33	46,XY,ider(20)(q10)del(20)(q11.2q13.1) [20]	20p13q13.13(70156_50932235) × 1 20q13.13q13.33(50934299_61861320) × 3	ins(11;?)(q23.3;?)/ KMT2A	exon10−exon2
34	46,XX,del(20)(q11.2q13.3)[1]/47,idem, +8[16] /46~47,idem,ins(8;11)(q24.2;q14q25)[cp3]	(8) × 3 20q11.21q13.31(33455109_57482326) × 1	ins(11;?)(q23.3;?)/ KMT2A	exon8−exon2
35	46,XX,del(7)(q21q36)[20]	7q21.3q36.2(96488457_154090770) × 1	ins(11;?)(q23.3;?)/ KMT2A	exon8−exon2
36	46,XY,del(20)(q11.2q13.3)[19]/46,XY [1]	20q11.21q13.13(32241971_51006470) × 1	dup(11)(q23.3q23.3)/ KMT2A	NA
37	46,XY [20]	No	ins(11;?)(q23.3;?)/ KMT2A ins(21;?)(q22.12;?)/ RUNX1	exon8−exon2
38	46,XX,del(5)(q14q33)[1]/46,idem,der(7)t(7;12)(q22;q21)del(12)(q21q22),der(12)inv(12)(p12q12)t(7;12)(q22;q21)[19]	5q14.3q33.2(89805168_155246009) × 1 7q22.2q22.3(104860533_106117224) × 1 7q35q36.1(146173208_149417208) × 1 12p13.2p12.2(11529351_21072975) × 1 12q21.1q21.31(74918720_81220056) × 1	ins(11;?)(q23.3;?)/ KMT2A	exon10−exon2
39	46,XY [20]	12q21.2q23.1(77212558_96570626) × 1	ins(11;?)(q23.3;?)/ KMT2A	NA
40	46,XX,der(16)t(1;16)(q21;q22)[3]/46,XX [17]	No	ins(11;?)(q23.3;?)/ KMT2A	NA
41	46,XY [20]	No	ins(11;?)(q23.3;?)/ KMT2A	exon8−exon2
42	46,XX [20]	No	ins(11;?)(q23.3;?)/ KMT2A	exon8−exon2
43	46,XY [20]	No	ins(11;?)(q23.3;?)/ KMT2A	NA
44	46,XY,del(20)(q11.2q13.2)[20]	20q11.21q13.2(32497427_51733992) × 1	ins(11;?)(q23.3;?)/ KMT2A	NA
45	46,XY [20]	No	dup(11)(q23.3q23.3)/ KMT2A	NA

**Table 3:** Gene mutations detected in patients with *KMT2A*-PTD. AR: allele ratio (ratio of the mutant ITD allele to the wild-type allele; VAF: variant allele frequency.

Case	Gene Mutation and VAF	Nomenclature by HGSV and COSMIC-ID	FLT3 ITD VAF and AR
1	NF1 p.R1534* 20% NF1 p.P2310fs 28% U2AF1 p.S34F 37% BCOR1 p.Q1314* 73%	NF1 (NM_001042492.3):c.6927_6928insAG p.P2310fs* 10 NF1 (NM_001042492.3): c.4600C > T p.R1534* COSM24466 U2AF1 (NM_006758.3): c.101C > T p.S34F COSM166866 BCOR (NM_017745.6): c.3940C > T p.Q1314*	VAF 1% AR 0.01
2	ETV6 p.S139fs 41% IDH2 p.R172K 37%	ETV6 (NM_001987.5): c.416_417del p.S139fs* 14 IDH2 (NM_002168.4): c.515G > A p.R172K COSM33733	Negative
3	DNMT3A p.Y735C 45% RUNX1 p.A251fs 27% RUNX1 p.R162 94% SRSF2 p.P95_R102del 63%	DNMT3A (NM_022552.5): c.2204A > G p.Y735C COSM231560 RUNX1 (NM_001754.5): c.749_750insGGGGAGG p.A251fs RUNX1 (NM_001754.5): c.485G > A p.R162K COSM96546 SRSF2 (NM_003016.5): c.284_307del p.P95_R102del	VAF 2% AR 0.02
4	NRAS p.G13R 34%	NRAS (NM_002524.5): c.37G > C p.G13R COSM569	Negative
5	DNMT3A p.R882H 23%	DNMT3A (NM_022552.5): c.2645G > A p.R882H COSM52944	VAF 1% AR 0.01
6	FLT3 p.N676K 37% PHF6 p.T170fs 32% RUNX1 p.D93fs 37% WT1 p.L383fs < 5%	FLT3 (NM_004119.3): c.2028C > A p.N676K COSM303886 PHF6 (NM_032458.3): c.509delinsTT p.T170fs RUNX1 (NM_001754.5): c.276_277insTC p.D93fs WT1 (NM_024426.6): c.1145_1146insAAGAGCAGCGACGTGTGCCTGGAGTAGCCCCGAC p.L383fs	Negative
7	DNMT3A p.I780N 43% IDH1 p.R132C 39% STAG2 p.Q275* 31%	DNMT3A (NM_022552.5):c.2339T > A p.I780N IDH1 (NM_005896.4): c.394C > T p.R132C COSM28747 STAG2 (NM_006603.5): c.823C > T p.Q275*	Negative
8	ASXL1 p.G646fs 12% NRAS p.G13R < 5% PTPN11 p.D61Y < 5%	ASXL1 (NM_015338.6): c.1934dupG p.G646fs COSM34210 NRAS (NM_002524.5): c.37G > C p.G13R COSM569 PTPN11 (NM_002834.5): c.181G > T p.D61Y COSM13022	Negative
9	IDH1 p.R132S 30% STAG2 p.W315*, 63%	IDH1 (NM_005896.4): c.394C > A p.R132S COSM28747 STAG2 (NM_006603.5): c.945G > A p.W315*	Negative
10	DNMT3A p.R882C 40% IDH2 p.R140Q 42%	DNMT3A (NM_022552.5): c.2644C > T p.R882C COSM5304 IDH2 (NM_002168.4): c.419G > A p.R140Q COSM41590	VAF 66% AR 1.93
11	DNMT3A p.T834I 43% RUNX1 p.R207fs 45% SF3B1 p. p.G742D 42%	DNMT3A (NM_022552.5): c.2501C > T p.T834I COSM1169638 RUNX1 (NM_001754.5): c.619dupC p.R207fs SF3B1 (NM_012433.4): c.2225G > A p.G742D COSM145923	VAF 41% AR 0.69
12	RUNX1 p.S318fs 32% IDH2 p.R140Q 46%	RUNX1 (NM_001754.5):c.952dupT p.S318fs IDH2 (NM_002168.4): c.419G > A p.R140Q COSM41590	VAF 16% AR 0.19
13	DNMT3A p.Q816* <5% IDH2 p.R172K 14%	DNMT3A (NM_022552.5): c.2446C > T p.Q816* COSM99739 IDH2 (NM_002168.4):c.515G > A p.R172K COSM41295	Negative
14	ASXL1 p.G646fs 17% RUNX1 p.Y380* 48%	ASXL1 (NM_015338.6): c.1934dupG p.G646fs COSM34210 RUNX1 (NM_001754.5): c.1140C > A p.Y380*	Negative
15	IDH2 p.R140Q 46% JAK2 V617F <5% TET2 p. E1186* 42% TP53 p.R175G 94% WT1 p.F144fs 11%	IDH2 (NM_002168.4): c.419G > A p.R140Q COSM41590 JAK2 (NM_004972.4): c.1849G > T p.V617F COSM12600 TET2 (NM_001127208.3): c.3556G > T p.E1186* TP53 (NM_000546.6): c.523C > G p.R175G COSM10870 WT1 (NM_024426.6): c.4342_437delinsG p.F144fs	VAF 16% AR 0.2
16	TP53 p.R273C 41% U2AF1 p.S34F 45% NRAS p. Q61K 8% EVT6 p. p.N382fs* 41%	TP53 (NM_000546.6): c.817C > T p.R273C COSM10659 U2AF1 (NM_006758.3):c.101C > T p.S34F COSM166866 NRAS (NM_002524.5):c.181C > A p.Q61K COSM580 ETV6 (NM_001987.5): c.1145dupA p.N382fs*	VAF 5% AR 0.05
17	BCOR p.K1330* 66% DNMT3A p.R885W 36% KRAS p.G12D 47% U2AF1 p.S34F 48% NRAS p.G13R 7%	BCOR (NM_017745.6): c.3988A > T p.K1330* DNMT3A (NM_022552.5): c.2653A > T p.R885W COSM10075189 KRAS (NM_004985.5): c.35G > A p.G12D COSM521 U2AF1 (NM_006758.3): c.101C > T p.S34F COSM166866 NRAS (NM_002524.5): c.37G > C p.G13R COSM569	Negative
18	BCOR p.Y1373* 82% NF1 p.I679fs 33% TET2 p.S1898T 38% U2AF1 p.S34F 41%	BCOR (NM_017745.6.6): c.4118_4119del p.Y1373* NF1 (NM_001042492.3): c.2033dupC p.I679fs* 21 TET2 (NM_001127208.30): c.5692T > A p.S1898T U2AF1 (NM_006758.3): c.101C > T p.S34F COSM166866	Negative
19	BCOR p? 72% NRAS p.G12C 36% RUNX1 p.L98fs*24 36% TP53 p.C277F 8%	BCOR (NM_017745.6): c.4326 + 1G > A p.? NRAS (NM_002524.5): c.34G > T p.G12C COSM562 RUNX1 (NM_001754.5): c.292del p.L98fs* 24 TP53 (NM_000546.6): c.830G > T p.C277F COSM10749	Negative
20	DNMT3A p.R771* 48% DNMT3A p.R366H 47% U2AF1 p.S34F 36% FLT3 p.D835V <5%	DNMT3A (NM_022552.5): c.2311C > T p.R771* COSM231563 DNMT3A (NM_022552.5): c.1097G > A p.R366H COSM1169226 U2AF1 (NM_006758.3): c.101C > T p.S34F COSM166866 FLT3 (NM_004119.3): c.2504A > T p.D835V COSM784	Negative
21	TET2 p.C1396W 31% U2AF1 p.S34F 26%	TET2 (NM_001127208.3): c.4188C > G p.C1396W COSM211732 U2AF1 (NM_006758.3): c.101C > T p.S34F	Negative
22	DNMT3A p.G543C 40% SF3B1 p.G740V 36%	DNMT3A c.1627G > T p.G543C COSM87002 SF3B1 c.2219G > T p.G740V COSM6156130	VAF 34% AR 0.52
23	CBL splice site mutation FLT3 D835F 13%, FLT3 P.D835Y 30% PHF6 p.I314T 44% RUNX1 p.C108fs 41% STAG2 p.W485* 45%	CBL (NM_005188): c.1096-1G > C p.? FLT3 (NM_004119.3): c.2504A > T p.D835V COSM784 FLT3 (NM_004119.3):c.2503G > T p.D835Y COSM783 PHF6 (NM_032458.3): c.941T > C p.I314T COSM4385517 RUNX1 (NM_001754.5): c.320_321insGCTGGCG p.C108fs STAG2 (NM_006603.5): c.1455G > A p.W485*	VAF 45% AR 0.83
24	ASXL1 p.G642* < 5% DNMT3A p.P451fs 45% JAK2 p. V617F < 5% SRSF2 p.P95L 29% TET2 p.C1298Y 44% TET2 p.N488fs 42%	ASXL1 (NM_015338.6): c.1924G > T p.G642* COSM110710 DNMT3A (NM_022552.5): c.1352del p.P451fs* JAK2 (NM_004972.4): c.1849G > T p.V617F COSM12600 SRSF2 (NM_003016.5): c.284C > T p.P95L COSM146288 TET2 (NM_001127208.3): c.3893G > A p.C1298Y COSM87138 TET2 (NM_001127208.3): c.1461del p.N488fs	Negative
25	DNMT3A p.R882H 29% WT1 p.S386*, 12%	DNMT3A (NM_022552.5): c.2645G > A p.R882H COSM52944 WT1 (NM_024426.6): c.1157C > A p.S386* COSM27307	VAF 6% AR 0.06
26	STAG2 p.? 81% TET2 p.L1721fs 32% WT1 p.Y300* 27%	STAG2 (NM_006603.5): c.1822-1G > A p.? TET2 (NM_001127208.3): c.5162dupT p.L1721fs WT1 (NM_024426.6): c.900C > G p.Y300*	VAF 34% AR 0.51
27	DNMT3A p.M801V 40% TET2 p.Y437* 66%, TET2 p.N801fs 31% U2AF1 p.S34F 49%, CBL p.p417R < 5%	DNMT3A (NM_022552.5): c.2401A > G p.M801V COSM5944905 TET2 (NM_001127208.3):c.1311C > A p.Y437* TET2 (NM_001127208.3):c.2400_2415del p.N801fs U2AF1 (NM_006758.3): c.101C > T p.S34F COSM166866 CBL (NM_005188):c.1250C > G p.P417R COSM34081	VAF 84% AR 5.2
28	DNMT3A p.R 882C 36% PHF6 p.R274*, 41%, WT1 p.K464fs 48% WT1 p.H412fs 34% FLT3 p.V592D 45%	DNMT3A (NM_022552.5): c.2644C > T p. R882C; COSM53042 PHF6 (NM_032458.3): c.820C > T p.R274* WT1 (NM_024426.6): c.1390_1391 insGGGACTA p.K464fs WT1 (NM_024426.6): c.1235_1259 delinsCCG p.H412fs FLT3 (NM_004119.3): c.1775T > A p.V592D COSM5879551	Negative
29	ETV6 p.A377V 36% IDH2 p.R140Q 40% WT1 p.G186fs 83%	ETV6 (NM_001987.5):c.1130C > T IDH2 (NM_002168.4): c.419G > A; p.R140Q COSM41590 WT1 (NM_024426.6): c.555dupC p.G186fs	Negative
30	DNMT3A p. R882H 39% IDH2 p.R 140Q 47% TP53 p.R273H VAF 31%	DNMT3A (NM_022552.5): c.2645G > A p.R882H COSM52944 IDH2 (NM_002168.4): c.419G > A p.R140Q COSM41590 TP53 (NM_000546.6):c.818G > A p.R273H COSM10660	VAF 26% AR 0.35
31	DNMT3A p. R882H 38% BCOR p.Q1624fs* 99% WT1 p.S285fs 43%	DNMT3A (NM_022552.5): c.2645G > A p.R882H COSM52944 BCOR (NM_017745.6): c.4871_4872del p.Q1624fs* 13 WT1 (NM_024426.6): c.850_851dupGG p.S285fs* 7	VAF 74% AR 2.81
32	BCOR p.M461fs 91% DNMT3A p.R882C 94% IDH1 p.R132S 38% RUNX1 p.P201Q 50%	BCOR (NM_017745.6):c.1378_1379dupAA p. M461fs DNMT3A (NM_022552.5): c.2644C > T p.R882C IDH1 (NM_005896.4): c.394C > A p.R132S COSM28748 RUNX1 (NM_001754.5): c.602G > A p.P201Q COSM24805	Negative
33	TET2 p.R1660fs VAF 26% U2AF1 p.S34F VAF 42%	TET2 (NM_001127208.3): c.4979_4994del p.R1660fs U2AF1 (NM_006758.3): c.101C > T p.S34F COSM166866	Negative
34	ASXL1 p.Q768* 44% FLT3 p.N676K 22% IDH1 p.R132C 43% NF1 p.2558* 48% RUNX1 p.240Q 54% SRSF2 p.P95H 45% STAG2 p.Y578* 39% KRAS p.G12A 5%	ASXL1 (NM_015338.6): c.2302C > T p.Q768* COSM41717 FLT3 (NM_004119.3): c.2028C > G p.N676K COSM303886 IDH1 (NM_005896.4): c.394C > T p.R132C COSM28747 NF1 (NM_001042492.3): c.6772C > T p.R2258* RUNX1 (NM_001754.5): c.611G > A p.R204Q COSM24731 SRSF2 (NM_003016.5): c.284C > A p.P95H COSM144993 STAG2 (NM_006603.5): c.1734C > G p.Y578* KRAS c.35G > C p.G12A COSM522	Negative
35	TET2 p.C1211del 37% TET2 p.F519fs 26%	TET2 (NM_001127208.3):c.3632_3634del p.C1211del TET2 (NM_001127208.3): c.1557del p.F519fs* 14	VAF 12% AR 0.14
36	DNMT3A p. W297* 40% U2AF1 p.S34F 45%	DNMT3A (NM_022552.5): c.891G > A p.W297* U2AF1 (NM_006758.3): c.101C > T p.S34F COSM166866	Negative
37	DNMT3A p.R288C 35% FLT3 p.D839G < 5% U2AF1 p.S34F 49%	DNMT3A (NM_022552.5): c.2644C > T p.R882C COSM53042 FLT3 (NM_004119.3): T > C c.2516A > G p.D839G COSM1166729 U2AF1 (NM_006758.3): G > A c.101C > T p.S34F COSM166866	Negative
38	PTPN11 p.G503A 35% STAG2 p? 35% RUNX1 p.Q262fs < 5%	PTPN11 (NM_002834.5):c.1508G > C p.G503A COSM13027 STAG2 (NM_006603.5):c.1416G > T (splicing site) p.? RUNX1 (NM_001754.5):c.784C > T p.Q262* COSM270868	Negative
39	STAG2 p.T1122fs 42% RUNX1 p. N153fs 38% SRSF2 p. P95L 27% IDH2 p.R172K 5% ASXL1 p.G646fs 5%	STAG2 (NM_006603.5):c.3364_3365insAA p.T1122fs RUNX1 (NM_001754.5): c.456_457dupGA p.N153fs SRSF2 (NM_003016.5): c.284C > T p.P95L COSM146288 IDH2 (NM_002168.4): c.515G > A p.R172K COSM41295 ASXL1 (NM_015338.6): c.1934dupG p.G646fs COSM34210	Negative
40	DNMT3A p.R635W 38% IDH2 p.R140G 42% SF3B1 p.K700E 47%	DNMT3A (NM_022552.5):c.1903C > T p.R635W COSM87012 IDH2 (NM_002168.4): c.418C > G p.R140G COSM96477 SF3B1 (NM_012433.4): c.2098A > G p.K700E COSM133591	Negative
41	ASXL1 p.G6464fs 17% FLT3 p.A680V < 5% RUNX1 p.A292fs 43% NRAS p.Q61K < 5%	ASXL1 (NM_015338.6): c.1934dupG p.G646fs COSM34210 FLT3 (NM_004119.3): c.2039C > T p.A680V COSM786 NRAS (NM_002524.5): c.181C > A p.Q61K COSM580 RUNX1 (NM_001754.5): c.870_873dupCATT p.A292fs	Negative
42	DNMT3A p.R882H 37% FLT3 p.D835E < 5%	DNMT3A (NM_022552.5): c.2645G > A p.R882H COSM52944 FLT3 (NM_004119.3): c.2505T > G p.D835E COSM788	VAF 10% AR 0.11
43	DNMT3A p. R882C 36% NRAS p.Q61K 33%	DNMT3A (NM_022552.5): c.2644C > T p. R882C COSM53042 NRAS (NM_002524.5): c.181C > A p.Q61K COSM580	Negative
44	BCOR p.R1480* 81% NRAS p.G13V 35% U2AF1 p.34F 39% RUNX1 p.S48fs 52%	BCOR (NM_017745.6): c.4438C > T p.R1480* NRAS (NM_002524.5): c.38G > T p.G13V COSM574 U2AF1 (NM_006758.3):c.101C > T p.S34F COSM166866 RUNX1 (NM_001754.5): c.140dupT p.S48fs*90	Negative
45	ASXL1 p.G646fs 16% CBL p.C384F 29% FLT3 p.D835Y 7% KIT p.D816V 14% RUNX1 p.R162G 43%	ASXL1 (NM_015338.6): c.1934dupG p.G646fs* COSM34210 CBL (NM_005188): c.1151G > T p.C384F COSM34068 FLT3 (NM_004119.3):c.2503G > T p.D835Y COSM783 KIT (NM_000222.3):c.2447A > T p.D816V COSM1314 RUNX1 (NM_001754.5):c.484A > G p.R162G COSM24718	Negative

## Data Availability

The datasets used and/or analyzed during the current study are available from the corresponding author on reasonable request.

## Supplementary Materials

The following supporting information can be downloaded at: https://www.mdpi.com/article/10.3390/cancers16244193/s1, Figure S1: The Genome Browser illustrates various forms of KMT2A PTD, all labeled as ins(11q23;?) by OGM, differing in repeat number, size, and breakpoints. Repeated sequences are underlined. A (Case #38, MDS): OGM provides the coordinates RefStartPos 118,477,357 and RefEndPos 118,479,068, with a size 35,074 bp, and VAF of 0.39. Manual examination reveals a duplication with breakpoints within intron 1 and intron 5, spanning 5 labels. Table S1: Structural variants (insertion and duplication) detected by optical genome mapping, the breakpoints (RefStartPos, RefEndPos), length, VAF (variant allele frequency). * by manual exam; ** fractional copy number of KMT2A-PTD estimated based on VAF, number of repeats and the blast counts. Figure S2: OGM labels (corresponding to the CTTAAG motif) within KMT2A. A: The upper panel lists the 36 exons of KMT2A, while the lower panel shows the distribution of labels, with a higher concentration observed within intron 1. B: A higher magnification view focused on exons 2 to 17, highlighting an absence of labels between exons 6 and 16. B (Case #3, AML): OGM provides the coordinates RefStartPos 118,477,357 and RefEndPos 118,479,068, size 25,447 bp and VAF of 0.88. Manual examination reveals a duplication with breakpoints within intron 1 and intron 5, spanning 4 labels. C (Case #11, AML): OGM provides the coordinates RefStartPos 118,470,405 and RefEndPos 118,479,068, with a size of 16,189 bp and VAF of 0.97. Manual examination reveals a duplication with breakpoints within intron 2 and intron 5, spanning 3 labels. D (Case #42, CMML): OGM provides the coordinates RefStartPos 118,461,867 and RefEndPos 118,479,068, with a size of 47,562 bp and VAF of 0.82. Manual examination reveals a quadruple repeat with breakpoints within intron 1 and intron 5, spanning 3 labels.
