# Supplementary material for: Detection of KMT2A Partial Tandem Duplication by Optical Genome Mapping in Myeloid Neoplasms: Associated Cytogenetics, Gene Mutations, Treatment Responses, and Patient Outcomes
Source: Cancers (Basel). 2024 Dec 16;16(24):4193. doi: 10.3390/cancers16244193 (PMC11674272; doi:10.3390/cancers16244193)

Figure S1. The Genome Browser illustrates various forms of KMT2A PTD, all labeled as ins(11q23;?) by OGM, differing in repeat number, size, and breakpoints. Repeated sequences are underlined.

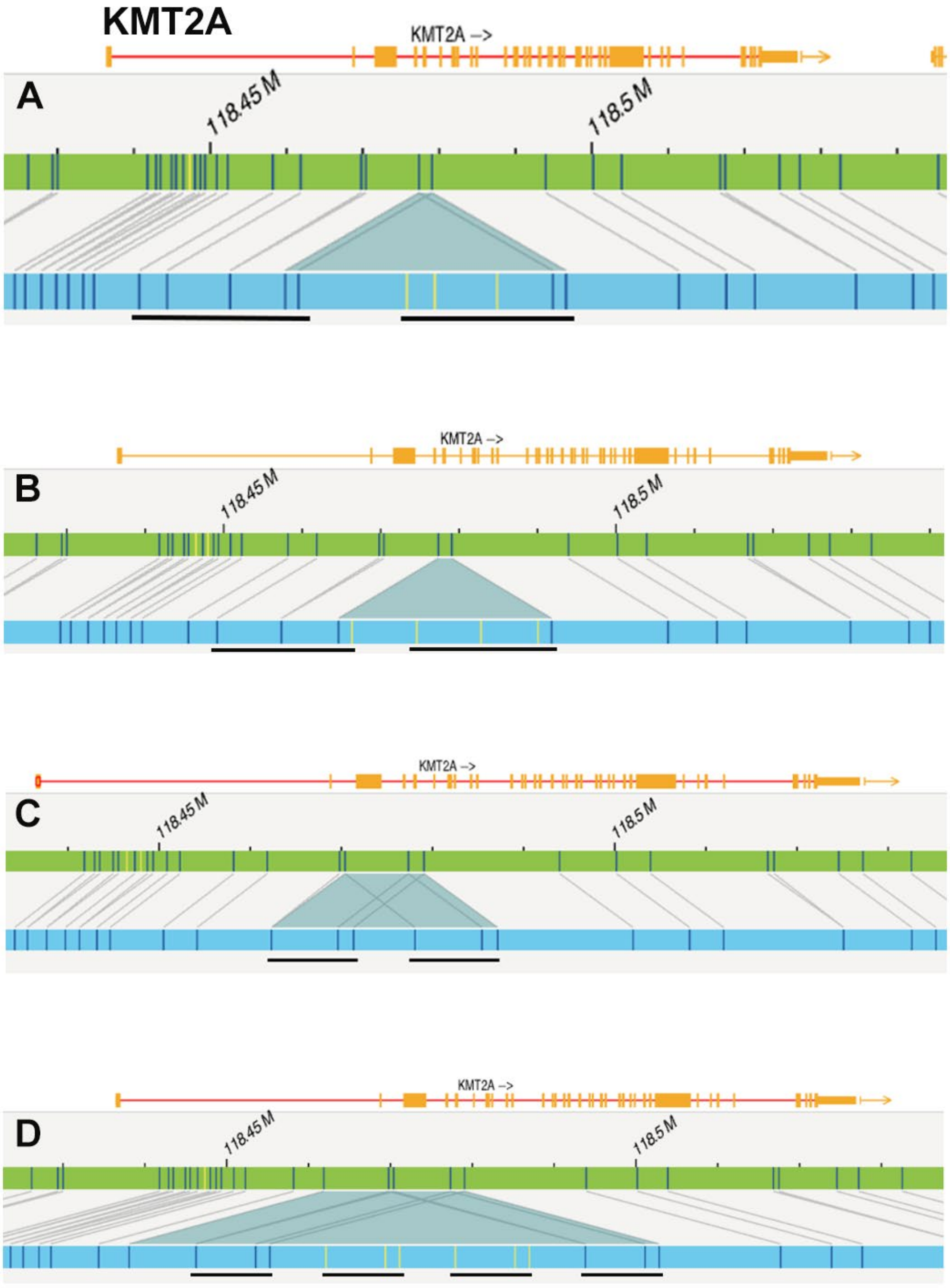

A (Case #38, MDS): OGM provides the coordinates RefStartPos 118,477,357 and RefEndPos 118,479,068, with a size 35,074 bp, and VAF of 0.39. Manual examination reveals a duplication with breakpoints within intron 1 and intron 5, spanning 5 labels.

B (Case #3, AML): OGM provides the coordinates RefStartPos 118,477,357 and RefEndPos 118,479,068, size 25,447 bp and VAF of 0.88. Manual examination reveals a duplication with breakpoints within intron 1 and intron 5, spanning 4 labels.

C (Case #11, AML): OGM provides the coordinates RefStartPos 118,470,405 and RefEndPos 118,479,068, with a size of 16,189 bp and VAF of 0.97. Manual examination reveals a duplication with breakpoints within intron 2 and intron 5, spanning 3 labels.

D (Case #42, CMML): OGM provides the coordinates RefStartPos 118,461,867 and RefEndPos 118,479,068, with a size of 47,562 bp and VAF of 0.82. Manual examination reveals a quadruple repeat with breakpoints within intron 1 and intron 5, spanning 3 labels.

Table S1. Structural variants (insertion and duplication) detected by optical genome mapping, the breakpoints (RefStartPos, RefEndPos), length, VAF (variant allele frequency). *by manual exam; **fractional copy number of *KMT2A*-PTD estimated based on VAF, number of repeats and the blast counts.

Cases	Abnormality	RefStartPos (bp)	RefEndPos (bp)	Length (bp)	VAF	Number of Repeats*	Fractional copy number**
1	ins(11;?)(q23.3;?)	118,477,357	118,479,068	16,516	0.86	2	4.7
2	ins(11;?)(q23.3;?)	118,479,068	118,493,942	11,368	0.78	2	5.2
3	ins(11;?)(q23.3;?)	118,477,357	118,479,068	25,447	0.88	2	4.0
4	ins(11;?)(q23.3;?)	118,470,405	118,479,068	32,625	0.85	3	17.0
5	ins(11;?)(q23.3;?)	118,461,867	118,479,068	31,007	0.78	3	9.0
6	ins(11;?)(q23.3;?)	118,470,405	118,479,068	32,090	0.67	3	11.5
7	ins(11;?)(q23.3;?)	118,470,405	118,479,068	32,626	0.83	3	6.9
8	ins(11;?)(q23.3;?)	118,479,068	118,493,942	188,818	0.72	2	5.4
9	ins(11;?)(q23.3;?)	118,477,357	118,479,068	25,311	0.74	2	5.6
10	ins(11;?)(q23.3;?)	118,470,405	118,479,068	27,729	0.86	3	9.7
11	ins(11;?)(q23.3;?)	118,470,405	118,479,068	16,489	0.93	2	8.6
12	ins(11;?)(q23.3;?)	118,470,405	118,479,068	32,077	0.79	3	6.5
13	ins(11;?)(q23.3;?)	118,470,405	118,479,068	28,383	0.88	2	7.3
14	ins(11;?)(q23.3;?)	118,479,068	118,493,942	28,677	0.81	2	8.8
15	ins(11;?)(q23.3;?)	118,479,068	118,479,068	26,815	0.73	2	3.5
16	ins(11;?)(q23.3;?)	118,461,867	118,479,068	30,123	0.86	3	11.0
17	ins(11;?)(q23.3;?)<br>ins(21;?)(q22.12;?)	118,479,068<br>34,880,853	118,479,068<br>34,902,218	15808<br>23,906	0.7<br>0.35	2<br>2	8.0
18	ins(6;?)(q23.3;?)<br>dup(11)(q23.3q23.3)<br>dup(21)(q22.2q22.2)	135,193,068<br>118,448,714<br>38,341,143	135,198,032<br>118,479,068<br>38,593,357	12,232<br>30,354<br>252,214	0.44<br>0.79<br>1.0	2<br>2<br>2	7.5
19	ins(11;?)(q23.3;?)	118,479,068	118,493,942	24,640	0.77	2	3.5
20	ins(11;?)(q23.3;?)<br>dup(21)(q22.2q22.2)	118,461,867<br>38,303,022	118,479,068<br>38,507,908	37,307	0.42	3	5.6
21	ins(11;?)(q23.3;?)	118,479,068	118,493,942	20,127	0.85	2	17.0
22	ins(11;?)(q23.3;?)	118,470,405	118,479,068	39,517	0.83	3	7.2
23	ins(11;?)(q23.3;?)	118,470,405	118,479,068	23,160	0.84	2	3.5
24	ins(11;?)(q23.3;?)	118,470,405	118,479,068	23,160	0.37	4	3.0
25	ins(11;?)(q23.3;?)	118,470,405	118,479,068	19,565	0.93	2	8.1
26	ins(11;?)(q23.3;?)	118,470,405	118,479,068	32,594	0.79	3	5.7
27	ins(11;?)(q23.3;?)	118,470,405	118,479,068	21,127	0.79	3	5.3
28	ins(11;?)(q23.3;?)	118,477,357	118,479,068	32,780	0.87	3	5.9
29	ins(11;?)(q23.3;?)	118,470,405	118,479,068	32,193	0.76	3	5.1
30	ins(11;?)(q23.3;?)	118,477,357	118,479,068	24,606	0.86	3	5.9
31	ins(11;?)(q23.3;?)	118,470,405	118,479,068	31,999	0.85	3	5.5
32	dup(11)(q23.3q23.3)	118,448,714	118,479,068	30,354	0.86	2	14.3
33	ins(11;?)(q23.3;?)	118,470,405	118,479,068	23,352	0.73	2	17.2
34	ins(11;?)(q23.3;?)	118,470,405	118,479,068	18,645	0.89	2	4.0
35	ins(11;?)(q23.3;?)	118,470,405	118,479,068	32,052	0.68	3	4.9
36	dup(11)(q23.3q23.3)	118,448,714	118,479,068	30,354	0.79	2	NA

37	ins(11;?)(q23.3;?)<br>ins(21;?)(q22.12;?)	118,470,405<br>34,858,511	118,479,068<br>34,860,175	16,517<br>68,875	0.83<br>0.23	2<br>2	NA
38	ins(11;?)(q23.3;?)	118,470,405	118,479,068	16,517	0.83	2	NA
39	ins(11;?)(q23.3;?)	118,477,357	118,479,068	7,849	0.63	2	NA
40	ins(11;?)(q23.3;?)	118,477,357	118,479,068	7,843	0.83	2	NA
41	ins(11;?)(q23.3;?)	118,470,405	118,479,068	32,080	0.84	3	NA
42	ins(11;?)(q23.3;?)	118,461,867	118,479,068	47,562	0.82	4	NA
43	ins(11;?)(q23.3;?)	118,470,405	118,479,068	17,221	0.80	2	NA
44	ins(11;?)(q23.3;?)	118,469,796	118,477,357	15,187	0.77	2	NA
45	dup(11)(q23.3q23.3)	118,448,714	118,479,068	30,354	0.88	2	NA

Figure S2. OGM labels (corresponding to the CTTAAG motif) within *KMT2A*.

A: The upper panel lists the 36 exons of *KMT2A*, while the lower panel shows the distribution of labels, with a higher concentration observed within intron 1.

B: A higher magnification view focused on exons 2 to 17, highlighting an absence of labels between exons 6 and 16.

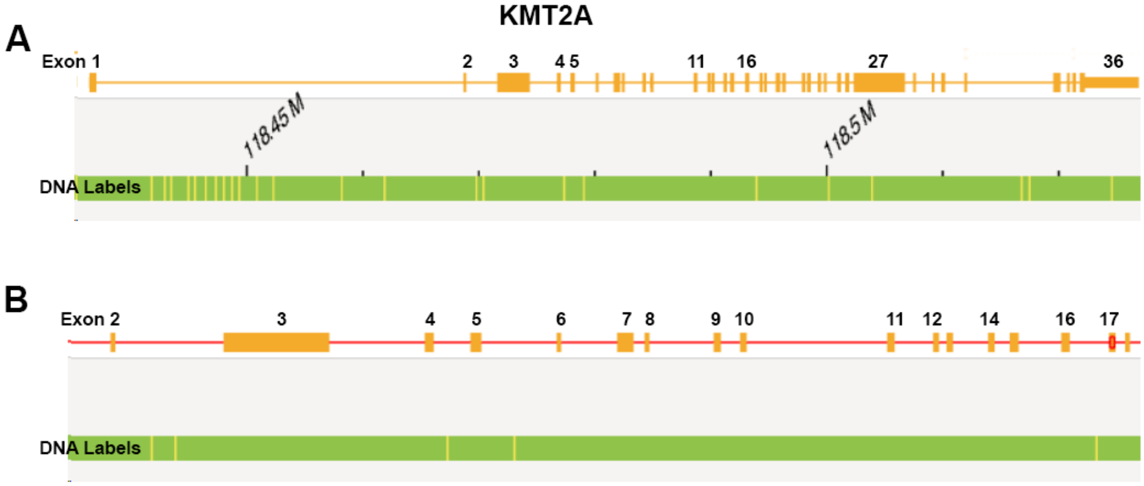

Supplement: Supplementary file 1 [file cancers-16-04193-s001.zip › cancers-3330919-supplementary.pdf]
